# L-carnitine does not improve valproic acid poisoning management: a cohort study with toxicokinetics and concentration/effect relationships

**DOI:** 10.1186/s13613-022-00984-z

**Published:** 2022-01-29

**Authors:** Philippe Nguyen, Lucie Chevillard, Ahmed S. Gouda, Hervé Gourlain, Laurence Labat, Isabelle Malissin, Nicolas Deye, Sebastian Voicu, Bruno Mégarbane

**Affiliations:** 1 Department of Medical and Toxicological Critical Care, Federation of Toxicology APHP, Lariboisière Hospital, 2 Rue Ambroise Paré, 75010 Paris, France; 2 University of Paris, Inserm UMRS-1144, Paris, France; 3 National Egyptian Center for Toxicological Researches, Faculty of Medicine, Cairo University, Cairo, Egypt; 4 Laboratory of Toxicology, Lariboisière Hospital, Paris, France

**Keywords:** Valproic acid, Poisoning, L-carnitine, Antidote, Pharmacokinetics, Lactate

## Abstract

**Background:**

Valproic acid (VPA) poisoning is responsible for life-threatening neurological and metabolic impairments. Despite only low-level evidence of effectiveness, L-carnitine has been used for years to prevent or reverse VPA-related toxicity. We aimed to evaluate the effects of L-carnitine used to treat acute VPA poisoning on the time-course of plasma VPA concentrations and VPA-related toxicity. We designed a single-center cohort study including all VPA-poisoned patients admitted to the intensive care unit. We studied VPA toxicokinetics using a nonlinear mixed-effects model-based population approach and modeled individual plasma VPA/blood lactate concentration relationships. Then, we evaluated L-carnitine-attributed effects by comparing VPA elimination half-lives and time-courses of blood lactate levels and organ dysfunction [assessed by the Sequential Organ Failure Assessment (SOFA) score] between matched L-carnitine-treated and non-treated patients using a multivariate analysis including a propensity score.

**Results:**

Sixty-nine VPA-poisoned patients (40F/29 M; age, 41 years [32–47]) (median [25th–75th percentiles]; SOFA score, 4 [1–6]) were included. The presumed VPA ingested dose was 15 g [10–32]. Plasma VPA concentration on admission was 231 mg/L [147–415]. The most common manifestations were coma (70%), hyperlactatemia (3.9 mmol/L [2.7–4.9]) and hyperammonemia (127 mmol/L [92–159]). VPA toxicokinetics well fitted a one-compartment linear model with a mean elimination half-life of 22.9 h (coefficient of variation, 28.1%). Plasma VPA (C)/blood lactate concentration (E) relationships were well described by an exponential growth equation [$E={E}_{0}\times {e}^{k\cdot C}$; with baseline *E*_*0*_ = 1.3 mmol/L (43.9%) and rate constant of the effect, *k* = 0.003 L/mg (59.5%)]. Based on a multivariate analysis, peak blood lactate concentration was the only factor independently associated with L-carnitine administration (odds ratio, 1.9, 95% confidence interval, 1.2–2.8; *P* = 0.004). We found no significant contribution of L-carnitine to enhancing VPA elimination, accelerating blood lactate level normalization and/or preventing organ dysfunction.

**Conclusions:**

VPA poisoning results in severe toxicity. While L-carnitine does not contribute to enhancing VPA clearance, its impact on accelerating blood lactate level normalization and/or preventing organ dysfunction remains uncertain. Investigating VPA toxicokinetics and concentration/effect relationships may help understanding how to improve VPA-poisoned patient management.

**Supplementary Information:**

The online version contains supplementary material available at 10.1186/s13613-022-00984-z.

## Background

Sodium valproate (VPA), a synthetic 2-propylpentanoic acid, is widely used as an antiepileptic, mood-stabilizer, antipsychotic, anti-migraine and analgesic drug [1]. VPA poisoning may on occasion cause severe toxicity and can very rarely be fatal. Based on the American Association of Poison Control Centers’ National Poison Data System, ~ 7,750 exposures to VPA including 119 severe and 2 fatal cases were reported in 2019 [2].

VPA poisoning is responsible for central nervous system manifestations ranging from ataxia, sedation and lethargy to coma, respiratory depression, seizures and intracranial hypertension [3]. Various metabolic disorders have been observed including hyperlactatemia and hyperammonemia attributed to VPA-induced alterations in mitochondria functions. Cases with cardiovascular, respiratory, hematological and/or liver failure have been reported and fatalities attributed to brain edema and multi-organ failure [4]. Peak plasma VPA concentration > 450 mg/L was likely associated with toxicity, while peak VPA > 850 mg/L with coma, respiratory depression, aspiration and lactic acidosis [5].

In general, VPA-poisoned patients are managed with supportive care alone and gastrointestinal decontamination, if appropriate [6]. Extracorporeal treatment to enhance VPA elimination, preferentially using intermittent hemodialysis, is only recommended in the most severe cases [7, 8]. Guidelines advise administering L-carnitine in the presence of coma, hyperammonemia and hyperlactatemia attributed to VPA-related toxicity and/or if VPA concentrations are > 850 mg/L [9]. However, despite its safety and excellent tolerance, evidence to support L-carnitine effectiveness in VPA overdose is scarce relying on anecdotal cases and small case-series [10, 11]. In the acute poisoning setting, no data support the ability of L-carnitine to alleviate or reverse VPA-induced central nervous system and liver dysfunction [12].

Randomized control trials are needed to evaluate the efficacy and safety of L-carnitine as an antidote, but their feasibility has been questioned due to the rarity of acute VPA poisoning. Therefore, since little is known about L-carnitine-related benefits limiting acute VPA toxicity, we designed this study in VPA-poisoned patients 1—to describe VPA toxicokinetics; 2—to analyze the relationships between plasma VPA concentrations and VPA-related effects on lactatemia; and 3—to evaluate L-carnitine effects on VPA elimination, blood lactate level normalization, and organ failure progress.

## Methods

### Study design

We conducted an 18-year single-center cohort study (2002–2020). The study was conducted according to Helsinki principles, declared to the *Commission Nationale de l’Informatique et des Libertés* (declaration number, 2067659) and approved by the ethics committee of the French Society of Intensive Care (protocol number, FICS20020231). During ICU stay, appropriate information was given to the patients and next of kin. Written informed consent was waived, since no specific intervention related to the research was performed.

### Patient selection and data collection

All VPA-poisoned adults consecutively admitted to our intensive care unit (ICU) with compatible history and features and at least one plasma VPA concentration > 100 mg/L (therapeutic range, 40–100) were included. Patients with missing medical records and patients with no available plasma VPA concentration to confirm the diagnosis of VPA poisoning were excluded. Past and recent medical history, comorbidities, presumed VPA ingested doses, co-ingested compounds, clinical presentation, laboratory parameters, toxicological analysis, management, complications and outcome were recorded. The Simplified Acute Physiology Score (SAPS) II [13] was determined on admission. The Sequential Organ Failure Assessment (SOFA) score [14] was calculated on admission and each day during the five following days. Acute kidney injury was graded according to the KDIGO classification [15].

### Patient management

VPA-poisoned patients were managed according to standards of care [9]. Physicians in charge decided plasma sampling to measure plasma VPA concentrations, treatments and L-carnitine dose regimen, which included an intravenous 100 mg/kg loading dose followed by a maintenance dosing up to 3 g/day in 3 divided doses for 3 days (or until ICU discharge). L-carnitine was administered based on the recommendations, which did not change during the study period.

### Toxicological analyses

Routine urine and plasma toxicological screening were obtained on ICU admission as performed in all our patients to identify the toxicants to which the patient was exposed. Plasma VPA concentrations were determined using a particle-enhanced turbidimetric immunoassay (Alinity c Valproic Reagent Kit®, Abbott, Prague, Check Republic; limit of detection, 0.8 µg/mL; limit of quantification, 6 µg/mL; limit of linearity, 150 µg/mL). Based on the declared information and toxicological screening results, plasma concentrations of co-ingested toxicants were determined using adequate quantitative assays if available in our institution.

### Toxicokinetic modeling

A population toxicokinetic analysis was performed using a nonlinear mixed-effects model. Only patients with available ingested dose, time from ingestion to admission and at least 2 plasma VPA concentrations were considered. Data were analyzed using NONMEM® version 6.2 (ICON Development Solutions, Ellicott City, MD). The first-order conditional estimation with interaction method was applied. A one-compartment linear model with linear absorption was used to describe VPA concentration–time profiles after oral exposure, as defined by the following equation:

1 $$C\left(t\right)=\frac{{k}_{a} \cdot PID \cdot F}{Vd \cdot \left(\frac{Cl}{Vd}-{k}_{a}\right)} \cdot \left({e}^{-{k}_{a} \cdot t}-{e}^{-\frac{Cl}{Vd} \cdot t}\right)$$

where *C*(t) is plasma VPA concentration; *k*_a_, the first-order absorption rate constant; PID, the presumed ingested dose; *F*, the bioavailability after oral exposure; *V*_d_ the volume of distribution; and Cl, the total body clearance.

Inter-individual variability was assumed exponential, and the full covariance matrix of the random effects was estimated. Additive, proportional, or mixed error models were tested to describe residual variability. The effects of each patient covariate (i.e., gender, age and L-carnitine treatment) on the toxicokinetic parameters were systematically tested.

The likelihood ratio based on the objective function value was used to test different hypotheses regarding the structure of the variance–covariance matrix for inter-individual variability and residual variability models, and to assess the covariate effects on toxicokinetic parameters.

For evaluation of goodness of fit, we obtained the following three graphs, i.e., population predicted versus observed concentrations, weighted residuals versus time and weighted residuals *versus* predicted VPA concentrations. Similar graphs using individual predictions were displayed. The population pharmacokinetic parameters are expressed as mean (coefficient of variation).

### Concentration-effect relationship modeling

We investigated VPA-attributed effects on blood lactate level in every individual. Only patients with at least five VPA/lactate paired values were considered. The relationship of blood lactate level (E) as function of plasma VPA concentration (C) estimated from Eq. 1 was described using the exponential growth equation:

2 $$E = {E}_{0}\times {e}^{k\cdot C}$$

where E_0_ is the baseline blood lactate level and k, the rate constant of the effect. The model was applied to VPA-attributed effects using the maximum likelihood expectation maximization algorithm implemented in WinNonlin® version 8.02 (Pharsight Corp., Mountain View, CA). All model parameters were assumed log-normally distributed. The model selection was based on goodness-of-fit criteria, which included the convergence criterion, the Akaike information criterion, the estimation criterion value for the maximum likelihood method, and the visual inspection of predicted *versus* observed and residual plots. Parameters of the concentration-effect relationships are expressed as mean (coefficient of variation) of individual estimations.

### Endpoint definitions

To evaluate L-carnitine effectiveness, we determined the three following endpoints in each patient: (1) VPA elimination half-life (*t*_1/2_); (2) the time from admission to normalize blood lactate level; and (3) the deltaSOFA defined as the difference between the worst SOFA score during the first 5 days of ICU stay and the SOFA score on admission.

### Statistical analysis

The qualitative variables are expressed as percentages and the quantitative variables as median [25th–75th percentiles]. Comparisons were performed using Mann–Whitney and Fisher exact tests, as appropriate. To analyze L-carnitine-attributed effects, a propensity score to determine the probability for each patient to be treated with L-carnitine was constructed and used in the multivariate analysis. The following confounding factors, i.e., gender, age, blood lactate level on admission, plasma VPA concentration on admission and SOFA score on admission were included in the propensity score calculation. Subsequently, we searched for a relationship between L-carnitine treatment and each endpoint, i.e., the *t*_1/2,_ the time for blood lactate level normalization and the deltaSOFA. For each criterion, we performed univariate analyses followed by a multivariate analysis using a linear stepwise regression model, in which significant variables at the *P* = 0.20-threshold in the univariate analyses, were included. The propensity score was kept in the final model regardless of its significance levels. The odds ratio and its 95% confidence interval were calculated for every independent parameter associated with L-carnitine treatment. All analyses were carried out with bilateral hypotheses. The statistical analysis was performed using XLStats® 2017 software (Addinsoft, New York, NY). *P-values* < 0.05 were considered as significant.

## Results

### Poisoning presentation, management and outcome

Sixty-nine consecutive VPA-poisoned patients (40F/29 M; age, 41 years [32–47]; body-mass index, 24.2 kg/m^2^ [21.3–29.2]) were included. Among these patients, 49 (71%) were chronically treated with VPA, in relation to epilepsy (*n* = 29, 42%), bipolar disorder (*n* = 35, 51%) and psychotic disorder (*n* = 22, 32%). The presumed VPA ingested dose was 15.0 g [10.0–32.0], rarely as sustained release formulation (*n* = 8, 14%). Exposure resulted from multi-drug ingestion (*n* = 45, 74%), involving benzodiazepines (*n* = 27, 39%), ethanol (*n* = 10, 15%), antipsychotics (*n* = 13, 19%) and hypnotics (*n* = 10, 15%).

On ICU admission, consciousness impairment (Glasgow Coma Score, 6 [3–14]) represented the main clinical manifestation (Table 1). Elevations in blood lactate level (2.9 mmol/L [1.8–4.2], normal range, 1.0–2.0) and ammonia (96 μmol/L [62–132]; normal range, 14–38) represented the main laboratory alterations. Plasma VPA concentration was 231 mg/L [147–415]. The SOFA score was 4 [1–6].

**Table 1:** Clinical and laboratory parameters in 69 valproic acid-poisoned patients on admission to the intensive care unit and univariate comparisons according to L-carnitine administration

	All patients (n = 69)	Patients managed without L-carnitine (n = 50)	Patients managed with L-carnitine (n = 19)	*p*-value
*Demographics and medical history*				
Age (years)	41 [32–47]	40 [34–47]	40 [29–48]	0.70
Weight (kg)	69 [62–81]	68 [60–80]	68.0 [63–91]	0.96
Size (cm)	168 [162–173]	165 [160–170]	170 [160–174]	0.54
Body-mass index (kg/m^2^)	24.2 [21.3–29.2]	24 [31–22]	23 [19–26]	0.94
Gender (F/M)*,* *n* (%)	40 (58%) / 29 (42%)	29 (58%) / 21 (42%)	11 (58%) / 8 (42%)	1.00
Mood disorder, *n* (%)	35 (51%)	29 (58%)	6 (32%)	0.063
Epilepsy, n (%)	29 (42%)	21 (42%)	8 (42%)	1.00
Psychotic disorder*,* *n* (%)	22 (32%)	14 (28%)	8 (42%)	0.39
Metabolic pathology*,* *n* (%)	8 (12%)	7 (14%)	1 (5%)	0.43
Heart disease*,* *n* (%)	6 (9%)	5 (10%)	1 (5%)	1.00
Liver disease*,* *n* (%)	4 (6%)	4 (8%)	0 (0%)	0.57
Diabetes mellitus*,* *n* (%)	2 (3%)	0 (0%)	2 (10%)	0.07
Chronic renal failure*,* *n* (%)	2 (3%)	2 (4%)	0 (0%)	1.00
Long-term VPA treatment*,* *n* (%)	49 (71%)	34 (68%)	15 (79%)	0.55
Sustained release VPA*,* *n* (%)	8 (14%)	3 (7%)	5 (33%)	0.026
Co-intoxications*,* *n* (%)	45 (74%)	33 (75%)	12 (71%)	0.75
Presumed dose ingested (g)	15.0 [10.0–32.0]	15.6 [10.0–31.0]	10.0 [3.0–30.0]	0.91
*Clinical parameters on ICU admission*				
Glasgow coma score	6 [3–14]	6 [3–13]	8 [3–14]	0.24
Temperature (°C)	36.8 [32.2–37.3]	36.8 [36.2–37.5]	36.7 [36.1–37.2]	0.18
Systolic blood pressure (mmHg)	119 [110–133]	119 [110–133]	113 [101–130]	0.67
Diastolic blood pressure (mmHg)	64 [57–74]	64 [56–73]	66 [50–75]	**0.035**
Heart rate (/min)	94 [82–104]	93 [82–102]	95 [74–104]	0.11
Respiratory rate (/min)	19 [16–22]	19 [16–21]	20 [16–24]	0.48
*Laboratory parameters on ICU admission*				
Serum creatinine (µmol/L)	73 [65–86]	74 [62–87]	70 [61–80]	0.39
Arterial pH	7.39 [7.36–7.43]	7.39 [7.36–7.43]	7.37 [7.36–7.44]	0.55
HCO_3_^−^ (mmol/L)	23 [20–26]	23 [20–26]	22 [19–24]	**0.003**
PaO_2_/FiO_2_ (mmHg)	355 [262–467]	355 [281–470]	308 [105–450]	0.63
AST (IU/L)	27 [20–42]	25 [21–45]	24 [20–36]	0.43
ALT (IU/L)	19 [11–31]	19 [11–30]	17 [10–31]	0.84
Prothrombin index (%)	86 [77–94]	86 [78–94]	83 [72–91]	0.71
Bilirubin (UI/L)	7 [5–11]	6 [5–11]	7 [5–10]	0.17
White blood cells (G/L)	7.1 [5.4–9.3]	7.1 [5.4–9.4]	6.1 [4.8–8.5]	**0.027**
Platelets (G/L)	202 [168–251]	196 [163–251]	199 [184–234]	0.36
Hemoglobin (g/dL)	13.3 [12.2–14.4]	13 [12–14]	14 [12–16]	**0.043**
Blood lactate level (mmol/L)	2.9 [1.8–4.2]	2.7 [1.7–4.1]	3.3 [2.0–4.7]	**0.001**
Blood ammonia level (mmol/L)	96 [62–132]	83 [38–129]	96 [40–131]	0.30
Plasma VPA concentration (mg/L)	231 [147–415]	210 [143–358]	274 [174–607]	0.11
*Physiological scores on ICU admission*				
SOFA score	4 [1–6]	4 [2–6]	5 [1–5]	0.59
SAPS II	32 [20–41]	30 [18–41]	35 [23–40]	0.35
*Peak laboratory parameters*				
Peak blood lactate level (mmol/L)	3.7 [2.5–4.9]	3.5 [2.5–4.9]	4.6 [3.0–6.1]	0.002
Peak blood ammonia level (mmol/L)	127 [92–248]	124 [48–275]	227 [147–302]	0.19
Peak plasma VPA concentration (mg/L)	249 [150–398]	231 [147–365]	287 [174–779]	0.06
*Complications in the ICU*				
Coma*,* *n* (%)	48 (70%)	30 (60%)	18 (95%)	**0.007**
Seizures*,* *n* (%)	2 (3%)	2 (4%)	0 (0%)	1.00
Agitation*,* *n* (%)	12 (17%)	9 (18%)	3 (18%)	1.00
Brain edema*,* *n* (%)	2 (3%)	0 (0%)	2 (11%)	0.073
Hypotension*,* *n* (%)	20 (29%)	14 (28%)	6 (32%)	0.77
Tachycardia*,* *n* (%)	20 (29%)	13 (26%)	7 (37%)	0.39
Cardiovascular failure*,* *n* (%)	14 (20%)	8 (14%)	6 (32%)	0.41
Lactic acidosis*,* *n* (%)	17 (36%)	12 (38%)	5 (33%)	0.31
Liver cytolysis*,* *n* (%)	6 (9%)	4 (8%)	2 (11%)	0.66
Thrombocytopenia*,* *n* (%)	15 (22%)	9 (18%)	6 (32%)	0.33
Anemia*,* *n* (%)	15 (22%)	8 (16%)	7 (37%)	0.099
Disseminated intravascular coagulation	2 (3%)	1 (2%)	1 (5%)	0.48
Acute kidney injury*,* *n* (%)				0.10
Stage 1	4 (6%)	2 (4%)	2 (11%)	
Stage 2	7 (10%)	4 (8%)	3 (16%)	
Stage 3	1 (2%)	0 (0%)	1 (5%)	
Aspiration pneumonia*,* *n* (%)	23 (33%)	14 (28%)	9 (47%)	0.16
Hospital-acquired infection*,* *n* (%)	10 (15%)	6 (12%)	4 (21%)	0.45
*Treatments in the ICU*				
Activated charcoal	32 (46%)	20 (40%)	12 (63%)	0.11
Ventilation Non-invasive ventilation Invasive ventilation	11 (16%) 41 (59%)	10 (20%) 25 (50%)	1 (5%) 16 (84%)	0.091
Norepinephrine*,* *n* (%)	13 (19%)	7 (14%)	6 (32%)	0.16
Sedation*,* *n* (%)	32 (46%)	22 (44%)	10 (53%)	0.59
Hemodialysis*,* *n* (%)	2 (3%)	0 (0%)	2 (11%)	0.073
Transfusion*,* *n* (%)	2 (3%)	1 (2%)	1 (5%)	0.48
*Outcome, n (%)* Home Medical ward Psychiatry department Death	29 (43%) 7 (10%) 29 (43%) 3 (4%)	22 (45%) 5 (10%) 21 (43%) 1 (2%)	7 (37%) 2 (11%) 8 (42%) 2 (11%)	0.49
*Length of ICU stay (days)*	3 [2–5]	2 [2–5]	5 [2–6]	0.06

Significant *p*-values (< 0.05) are noted in bold

*ALT* alanine aminotransferase, *AST* Aspartate aminotransferase, *ICU* intensive care unit, *SAPS II* Simplified Acute Physiology Score II, *SOFA* Sequential Organ Failure Assessment, *VPA* valproic acid. Acute kidney injury was staged based on KDIGO classification

During ICU stay, the patients developed coma (*n* = 48, 70%), agitation (*n* = 12, 17%), seizures (*n* = 2, 3%) and brain edema evidenced by CT-scan (n = 2, 3%). Blood lactate and serum ammonia concentrations increased in 38% and 30% of the patients, peaking at 3.9 mmol/L [2.7–4.9] and 127 mmol/L [92–159], respectively. Plasma VPA concentration increased in 27% of the patients peaking at 248 mg/L [147–398] within the first 24 h from admission in almost all cases. Twenty-three (33%) patients developed aspiration pneumonia, 13 (19%) cardiovascular failure and 10 (15%) hospital-acquired infections. Hypoxemia (*n* = 52, 75%; PaO_2_/FiO_2_ ratio, 297 mmHg [162–389]), lactic acidosis (*n* = 17, 36%), mild anemia (*n* = 15, 22%), thrombocytopenia (*n* = 15, 22%), disseminated intravascular coagulation (*n* = 2, 3%) and liver cytolysis (*n* = 6, 9%) were observed. Acute kidney injury with mild elevation in median serum creatinine (81 μmol/L [67–102]) was classified as KDIGO stage 1 (*n* = 4, 6%), stage 2 (*n* = 7, 10%), and stage 3 (*n* = 1, 2%).

Overall, management included invasive mechanical ventilation (*n* = 41, 59%; prior to hospital transfer, *n* = 34, 49%), noninvasive mechanical ventilation (*n* = 2, 3%), mask oxygen (*n* = 9, 13%), activated charcoal (*n* = 32, 46%), sedation (n = 32, 46%), intravenous L-carnitine (*n* = 19, 28%, administered on ICU admission, once the diagnosis of VPA poisoning was established), norepinephrine (*n* = 13, 19%), blood transfusion (*n* = 2, 3%) and hemodialysis (*n* = 2, 3%). No adverse effect was attributed to L-carnitine administration. The length of ICU stay was 3 days [2–5]. Three patients (4%) died in the ICU. For additional descriptive data of particular subgroups, see Additional file 1: Table S1.

#### Population toxicokinetics

**Fig. 1:**
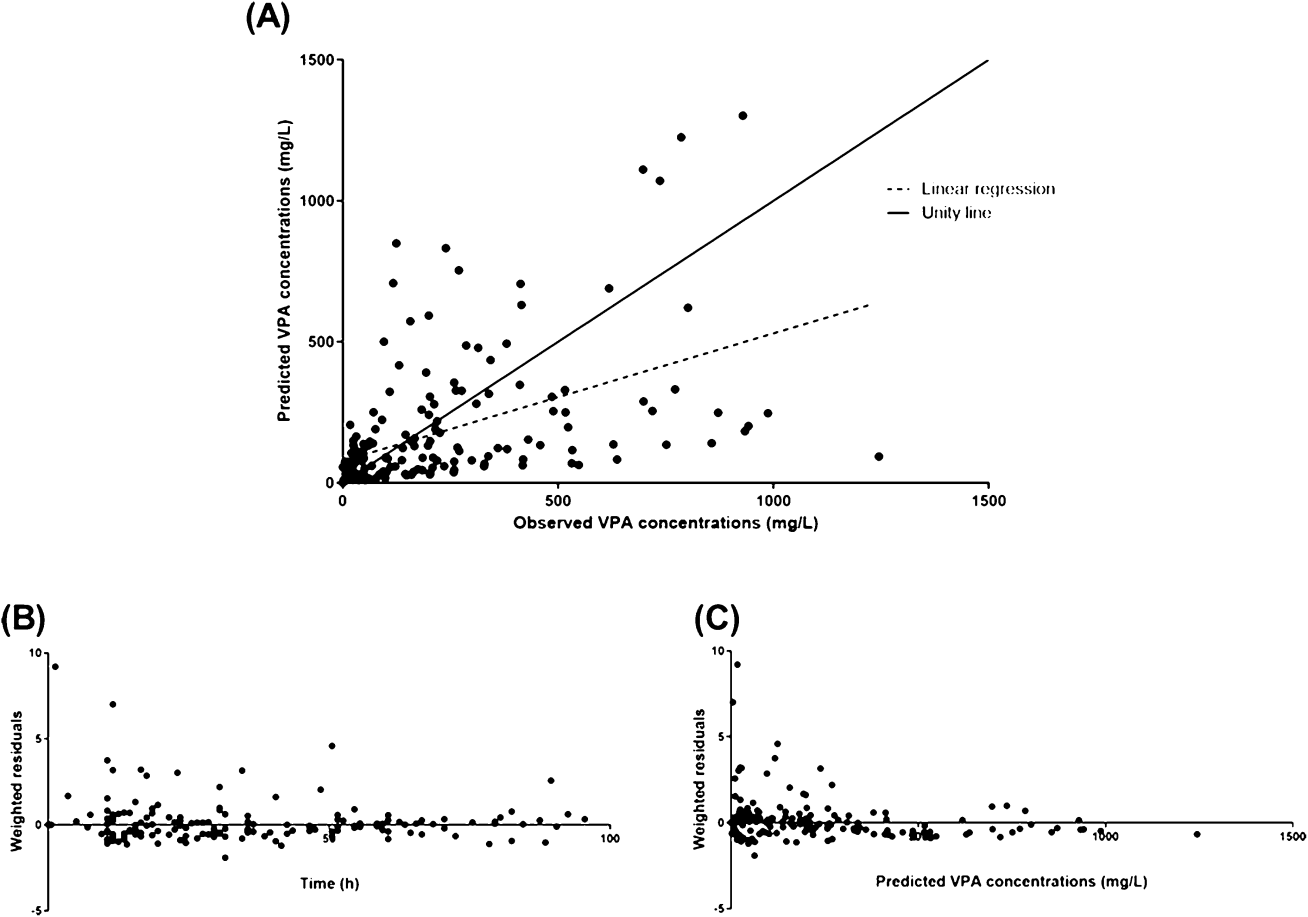
Validation of the valproic acid (VPA) toxicokinetic model in nineteen poisoned patients with the predicted *versus* observed plasma VPA concentrations (**A**), the weighted residuals *versus* time (**B**) and the weighted residuals *versus* predicted plasma VPA concentrations (**C**)

**Fig. 2:**
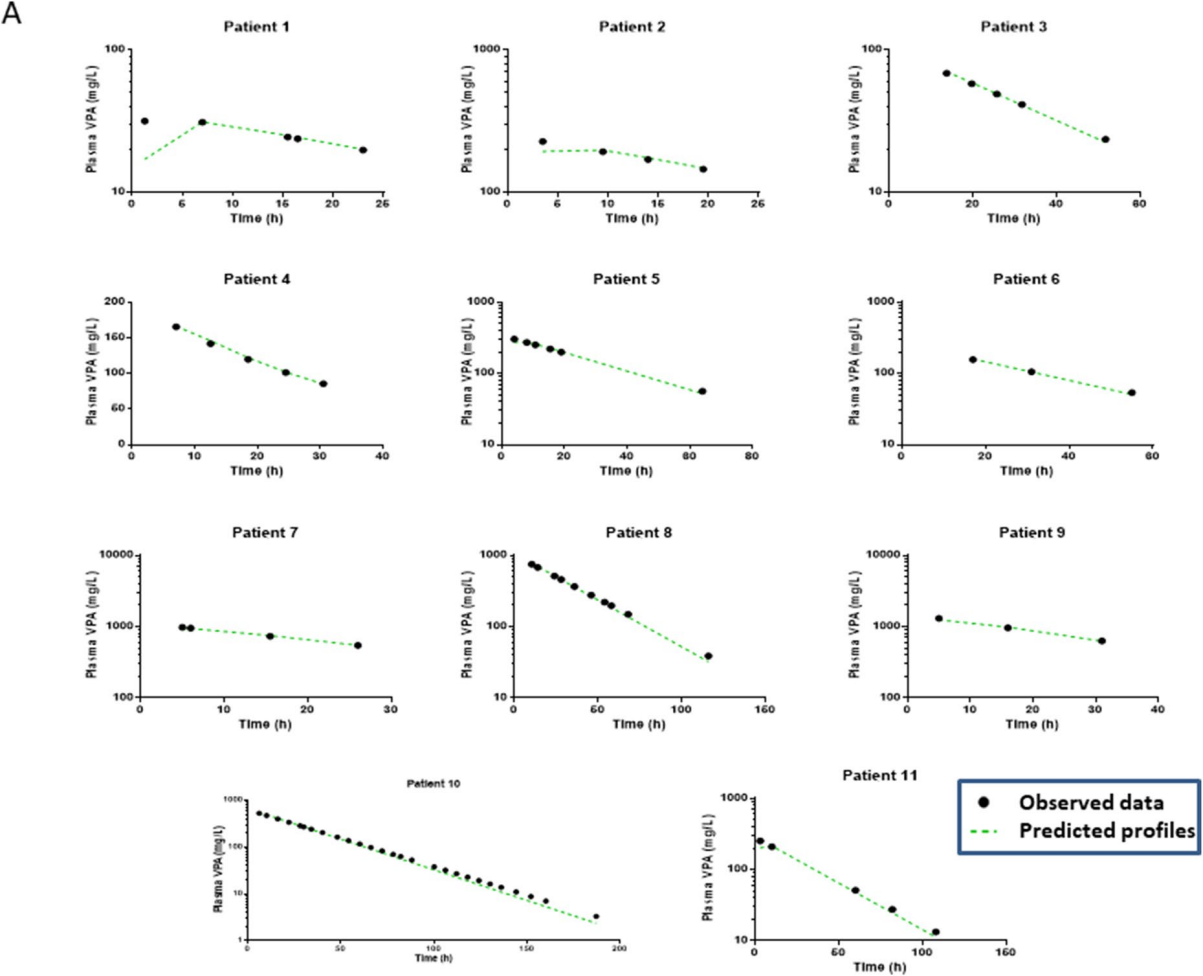

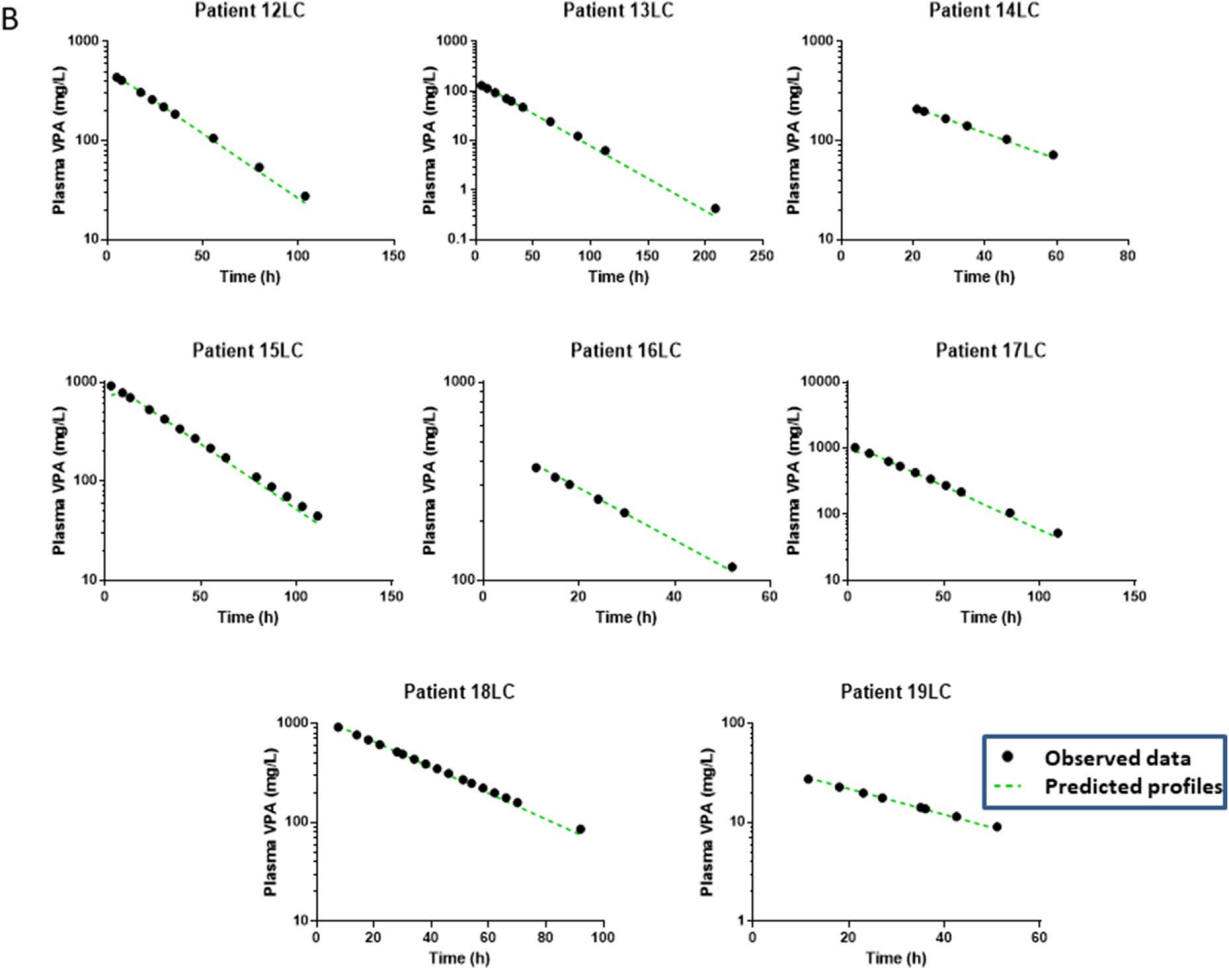
Individual toxicokinetics showing the best fit of the observed values in nineteen valproic acid (VPA)-poisoned patients non-treated (panel **A**) or treated with L-carnitine (designated as LC, panel **B**)

VPA toxicokinetics well fitted a one-compartment linear model with linear absorption (*n* = 19). The model parameters are presented in Table 2. The mean VPA *t*_1/2_ was 22.9 h (28.1%) and the mean apparent clearance 1.2 L/h (22.1%). The predicted *versus* observed VPA concentrations, the weighted residuals *versus* time and the weighted residuals *versus* predicted VPA concentrations are presented in Fig. 1. The individual model-predicted *versus* observed VPA concentrations are presented in Fig. 2.

**Table 2:** Parameters of the population toxicokinetic model of plasma valproic acid and parameters of the model of plasma valproic acid/blood lactate concentration relationships in valproic acid-poisoned patients managed in the intensive care unit

Parameters	Mean	CV%
*Population toxicokinetic model (n = 19)*		
k_a_ (/h)	0.5	Fixed
V_d_/F (L)	39.9	28.1
Cl/F (L/h)	1.2	22.1
ke (/h)	0.03	28.1
t_1/2_ (h)	22.9	28.1
η_(V/F)_ (%)	1.1	30.7
η_(Cl/F)_ (%)	0.9	32.3
ε_prop_	0.4	23.0
*Relationships between blood lactate and plasma valproic acid concentrations (n = 8)*		
E_0_ (mmol/L)	1.3	43.9
κ (L/mg)	0.003	59.5

k_a_, absorption rate constant; V_d_/F, apparent volume of distribution; Cl/F, apparent total body clearance; k_e_, elimination rate constant; t_1/2_, elimination half-life; η_(V/F)_, inter-individual variability in the apparent volume of distribution; η_(Cl/F)_, inter-individual variability in the apparent clearance; ε_prop_, proportional error of the model; E_0_, baseline blood lactate level; κ, rate constant of the effect; CV%, coefficient of variation

#### Plasma VPA/blood lactate concentration relationships

**Fig. 3:**
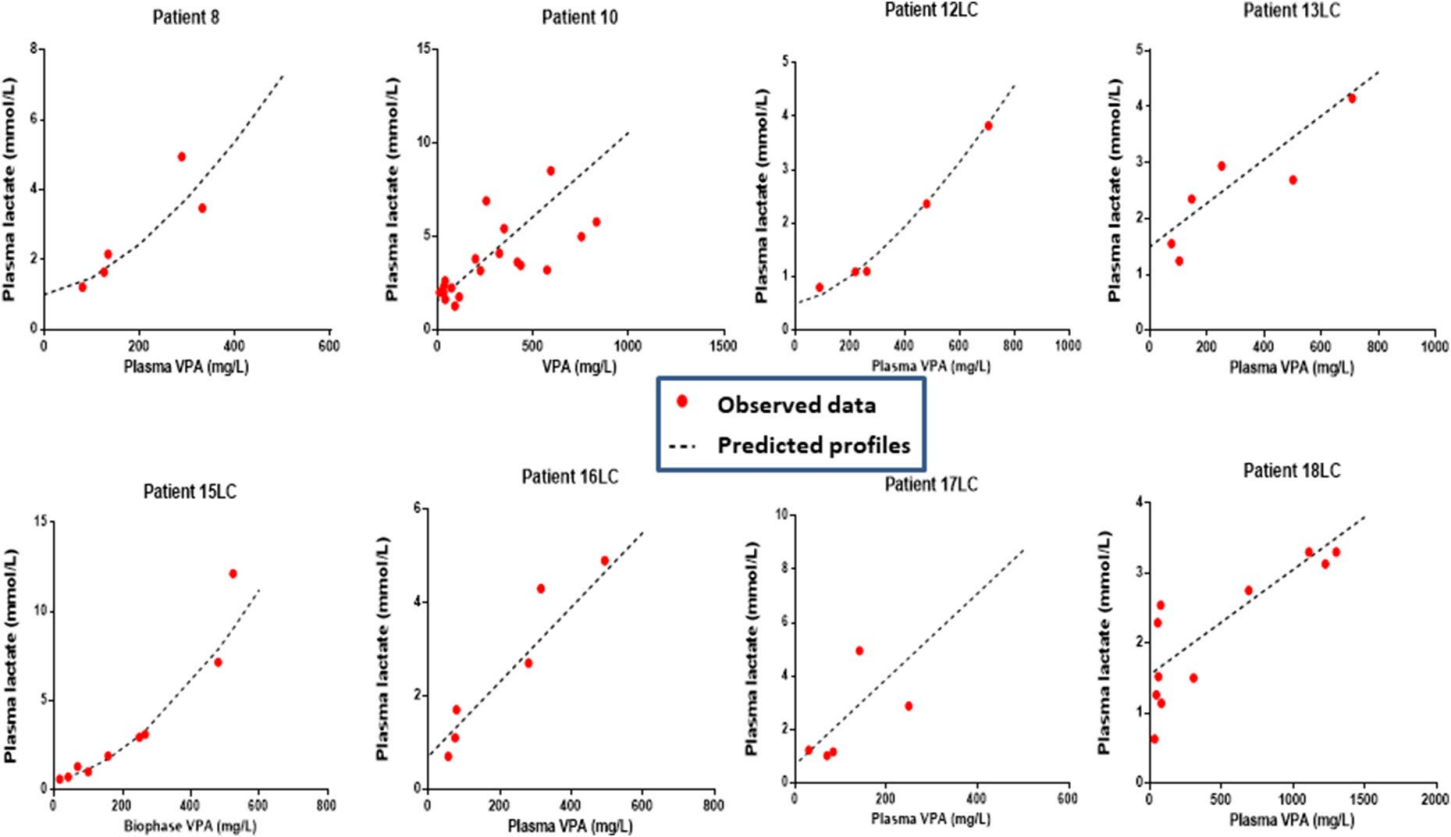
Individual relationships between blood lactate and plasma valproic acid (VPA) concentrations in eight valproic acid-poisoned patients. LC means treated with L-carnitine

**Fig. 4:**
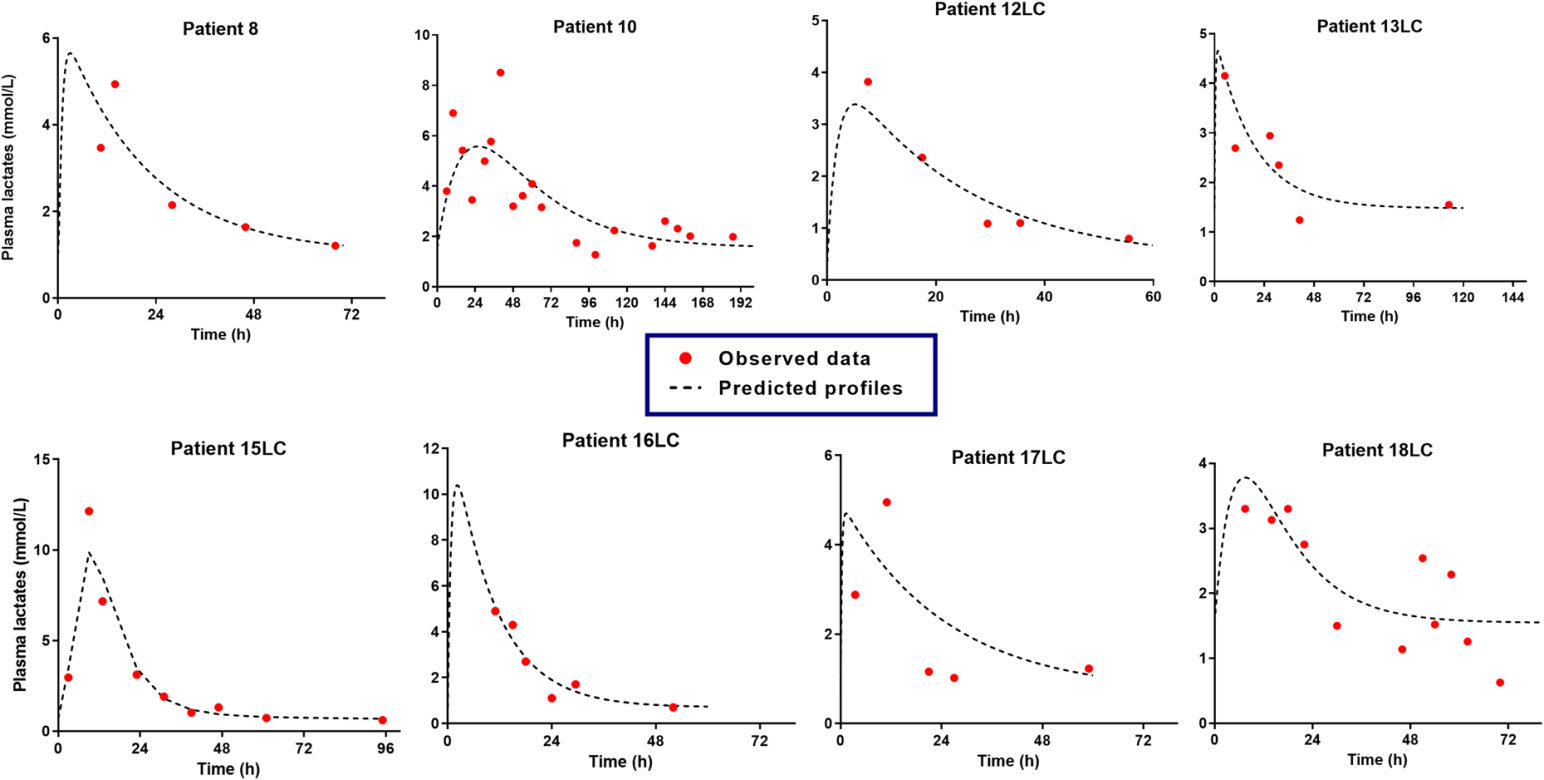
Individual time-course of blood lactate concentrations in eight valproic acid-poisoned patients. LC means treated with L-carnitine

The relationships between blood lactate and plasma VPA concentrations well fitted an exponential growth equation (n = 8). The model parameters are shown in Table 2. The model-predicted *versus* observed values are shown in Fig. 3. The time-course of VPA-induced effects on blood lactate concentrations are shown in Fig. 4.

#### Analysis of L-carnitine benefits in VPA poisoning

Based on univariate analyses, L-carnitine-treated and non-treated patients significantly differed regarding coma onset (95% *versus* 60%, *P* = 0.007), blood bicarbonate (22 mmol/L [19–24] *versus* 23 mmol/L [20–26], *P* = 0.003), blood lactate level (3.3 mmol/L [2.0–4.7] *versus* 2.7 mmol/L [1.7–4.1], *P* = 0.0013), hemoglobin (14 g/dL [12–16] *versus* 13 g/dL [12–14], *P* = 0.043), white blood cell count (6.1 G/L [4.8–8.5] versus 7.1 G/L [5.4–9.4], *P* = 0.027) (see more details in Table 1).

The construction of the propensity score is shown in Additional file 1: Table S2. In contrast to age, gender, SOFA score and VPA concentration on admission, the only parameter kept in the propensity score associated with L-carnitine administration was blood lactate level on admission (odds ratio, 1.31 [0.98–1.75], *P* = 0.068). Based on the multivariate analysis, the peak blood lactate level was the only parameter associated with L-carnitine administration (odds ratio, 1.9 [1.2–2.8]; *P* = 0.004; Additional file 1: Table S3).

In the univariate analyses, the delay to normalize blood lactate (21 h [13–24] versus 7 h [0–18], *P* = 0.03) differed between L-carnitine-treated and non-treated patients, whereas *t*_1/2_ (20 h [16–27] *versus* 18 h [12–25], *P* = 0.1) and the deltaSOFA (1.0 *versus* 1.0, *P* = 0.6) did not. In the multivariate analysis, no significant effect of L-carnitine administration was found on *t*_1/2,_ the delay to normalize blood lactate level and the deltaSOFA.

## Discussion

VPA poisoning is responsible for severe and even fatal presentations including coma, hyperlactatemia and hyperamonemia. L-carnitine administration was not associated with significant alteration in VPA elimination nor significant clinical or metabolic benefit.

### Mechanisms of VPA toxicity

Due to similar structures, VPA and medium-chain fatty acids are at risk of metabolic competition [4, 16, 17]. Multiple metabolic pathways are involved in VPA biotransformation, giving rise to ≥ 50 known metabolites. VPA undergoes mitochondrial β-oxidation and to a lesser extent microsomal ω-oxidation. Additional involved metabolic pathways include hydroxylation, glucuronidation and other minor conjugation reactions. VPA acyl-CoA esters formed in the cytosol by oxidation enter the mitochondria via the carnitine shuttle.

VPA-related toxicity results from the accumulation of some toxic metabolites, which may result from the misbalance between the intra-mitochondrial β-oxidation and the microsomal ω-oxidation, in relation to VPA transport blockage across the mitochondrial membrane [17]. These toxic metabolites contribute to the loss of mitochondrial membrane potential, modulate the activity of selected enzymes and transport systems, impair mitochondrial fatty acid β-oxidation and inhibit urea cycle [17, 18]. All these mitochondrial dysfunctions lead to VPA-related metabolic disorders including hyperammonemia, hyperlactatemia and liver microvesicular steatosis. Our findings clearly support relationships between lactate elevation and plasma VPA concentrations.

### Clinical consequences of VPA toxicity

In our series, the main VPA poisoning manifestation consisted of consciousness impairment, observed in 70% of the patients. However, severity of consciousness impairment in relation to the observed plasma VPA concentrations should be interpreted with cautious considering the multidrug ingestions mainly involving psychotropic drugs and the development of tolerance in the acute-on-chronically poisoned patients. Interestingly, we did not found significant differences when comparing acutely and acute-on-chronically VPA-poisoned patients, although we could not rule out an underpowered analysis. Two patients developed brain edema, which represents the most serious VPA-attributed neurological complication [3, 5]. Of note, in one of these two patients, edema occurred even though initial VPA concentration was moderately high (190 mg/L) but rapidly increased (up to 832 mg/L). This observation supports the necessity of repeating plasma VPA measurements despite initial reassuring values, due to prolonged absorption in overdose, especially with slow-release formulations. Both patients with brain edema were treated with L-carnitine and presented favorable outcome without neurological sequelae.

Interestingly, our findings also support a large interindividual variability regarding VPA-attributed effects on blood lactate concentrations as suggested by the elevated coefficients of variation of our pharmacodynamic model parameters (Table 2**).** However, the exact reasons for such a variability, which may correspond to patients with enhanced vulnerability to VPA toxicity, remains to be investigated.

### VPA toxicokinetics

Pharmacokinetics of VPA have been studied extensively, characterized by a high inter-individual variability [1]. In summary, gastrointestinal absorption is almost complete (≥ 80% bioavailability) allowing plasma concentration peaking before 2 or 8 h post-ingestion with the immediate *versus* sustained release formulation, respectively. Plasma protein binding is extensive (90–95%), increasing with age and decreasing at higher VPA concentrations. The volume of distribution ranges from 8.4 to 23.3 L using one-compartment models and 4.08 to 42.1 L using two-compartment models. VPA elimination follows first-order kinetics with a 0.206 to 1.154 L/h clearance. An average *t*_1/2_ of 10–12 h has been determined, with a range of 4–17 h. VPA undergoes extensive biotransformation, mainly in the liver, including mitochondrial β-oxidation (accounting for ≥ 40% of the dose and involving three cytochrome P450 enzyme isoforms CYP2C9, CYP2A6 and CYP2B6), other oxidative mechanisms (< 15–20%) and glucuronide conjugation (30–50%). Less than 5% of the ingested amount is eliminated unchanged in urine.

By contrast, very few toxicokinetic investigations exist. A population pharmacokinetic approach with Bayesian estimation, as performed in our VPA-poisoned patients, allows incorporating several factors affecting VPA kinetics into individualized drug therapy. In acute poisonings, absorption was shown to be rapid with peak concentrations observed 3.5–5.6 h post-ingestion [19]. VPA time-course was found biphasic, with *t*_1/2_ of 8.8–30.9 h and an apparent volume of distribution of 0.17–0.72 L/kg. The time-course of plasma VPA was studied later in another cohort of 20 VPA-poisoned patients using a two-compartment population pharmacokinetic model [20]. VPA therapy was shown to increase the V_max_ of β-oxidation by 59%. The observed differences in VPA elimination between the overdose and therapeutic settings were attributed to β-oxidation pathway saturation due to its Michaelis–Menten kinetics.

In our series, most of our patients were in the elimination phase on admission fitting a one-compartment linear model. This observation may in part be related to the fact that immediate-release formulations were predominantly ingested but may also support the benefits of activated charcoal administration including at repeated doses as carried out in almost half of our patients. Our *t*_1/2,_ clearance and volume of distribution were consistent with values found in overdose elsewhere [19, 20]. Our mildly prolonged *t*_1/2_ despite preserved clearance might correspond to the distribution volume increase in comparison to pharmacological conditions or to β-oxidation pathway saturation, as suggested [20]. Interestingly, gastrointestinal decontamination was shown to moderately lower the bioavailability by an average of 34% [20].

### Effectiveness of L-carnitine in VPA poisoning

Carnitine is an essential cofactor in VPA metabolism allowing its transport across the mitochondria membrane and permitting ammonia elimination. Hypocarnitinemia results from urine excretion of valproylcarnitine, decreased tubular reabsorption of carnitine and inhibition of endogenous carnitine production, thus increasing blood acyl-carnitine/free carnitine ratio [16, 18]. A lack of carnitine is thought to contribute to hyperammonemia and L-carnitine, the levorotatory form of carnitine, was suggested as possible treatment to lower ammonemia in long-term VPA-treated patients [21]. Experimental and clinical data suggest that early L-carnitine supplementation can improve the outcome of VPA-induced hepatotoxicity in chronically treated patients [18, 22]. L-carnitine supplementation effectively reversed VPA-induced hyperammonemic encephalopathy [23], despite the absence of correlation between its severity, plasma VPA and serum ammonia concentrations [16, 24]. Moreover, prophylactic supplementation had also been advocated in VPA-treated epileptic children at high-risk of toxicity [12]. By extension, because of its role in restoring beta-oxidation metabolism in the liver cell mitochondria, L-carnitine was proposed in severely VPA-poisoned patients, who present lactic acidosis, liver failure, brain edema, and/or VPA concentrations of > 850 mg/L.

In our department, the decision to administer L-carnitine was left to the physician in charge. The proportion of patients receiving L-carnitine did not change over time during the study period. Interestingly, we found that peak blood lactate was the only factor independently associated with L-carnitine infusion. This observation supported the fact that L-carnitine prescription in our ICU was consistent with the national guidelines [9]. We observed no side effects attributed to L-carnitine, consistent with previous reports establishing the excellent tolerance of L-carnitine, such as the study showing no adverse effects in relation to 251 L-carnitine doses administered in the setting of VPA toxicity [25].

Curiously, evidence to support effectiveness of L-carnitine in improving VPA poisoning is still poor. Reduction in *t*_1/2_ was attributed to L-carnitine in eight non-overdosed long-term VPA-treated patients (9.5 h versus 12 h, *P* < 0.05) [26]; however, *t*_1/2_ in this study were shorter compared to ours. A Romanian randomized controlled trial including 62 VPA-poisoned patients admitted to the ICU (28 treated *versus* 34 non-treated patients with L-carnitine 1,800 mg/day for 3 days) reported that L-carnitine reduced plasma VPA levels and facilitated the decrease in plasma ammonia concentrations [27]. However, this study was only published as a congress abstract without available additional descriptive data. Here based on a matched comparative study, we showed that L-carnitine administration was unable to accelerate VPA clearance, speed blood lactate level normalization or limit organ dysfunction. Our findings clearly suggest limiting L-carnitine administration to the most severe VPA-poisoned patients (such as patients with brain edema) and considering alternative therapies that more effectively alter VPA kinetics, such as hemodialysis [8] or meropemen infusion [28].

### Study limitations

Our study presents limitations. Its relatively limited sample size may have underpowered our comparative analyses. The prolonged study duration is a result of VPA poisoning rarity but similarly to the elevated prevalence of co-ingestions, may have introduced confounding factors. Nevertheless, our cases represent real life multidrug poisonings with possible non-identified co-ingested drugs based on the routine screening, risk of drug-drug interactions and no modifications in management with time. However, a case-by-case analysis of the involved toxicants in each patient did not show pertinent drug-drug interactions that could have altered VPA metabolism or elimination. In our approach based on the concentration-effect relationships, we did not account for all possible confounding factors known to influence lactate clearance. In the absence of marked cardiovascular, liver, and renal impairment on ICU admission, we considered that the time-course of blood lactate concentrations could be related almost exclusively to the time-course of VPA concentrations. Although two patients who developed VPA-related brain edema received L-carnitine and improved, no conclusion on its benefits on this major complication could be drawn. Interestingly, the activity and toxicity of the numerous VPA metabolites are poorly understood; none was measured in our study, as assays were not available in our laboratory. Finally, we acknowledge that although confirming all our other findings, the multivariate analysis of the effectiveness of L-carnitine in VPA poisoning should be interpreted with cautions given the number of variables entered in the model for a small number of outcomes. Likewise, we could not evaluate L-carnitine effects on plasma ammonia, which measurement was not readily available in our institution, thus limiting its repetitive determination. Therefore, due to such limitations, we strongly believe that a randomized placebo-controlled multicenter clinical study is warranted to definitively establish the exact role of L-carnitine in VPA poisoning. However, despite no evidence to support its benefits but due to its excellent tolerance and low cost, we believe that L-carnitine administration should still be considered in the meantime on a case-by-case basis, without postponing or substituting supportive care.

## Conclusion

VPA poisoning is responsible for life-threatening manifestations with mildly prolonged VPA elimination. Our findings suggest no benefits of L-carnitine administration on VPA clearance, hyperlactatemia resolution or organ function improvement in VPA-poisoned patients. However, the definitive evaluation of L-carnitine benefits requires a multicenter randomized controlled trial.

## Supplementary Information

**Additional file 1: Table S1.** Descriptive analysis of different subgroups of valproic acid-poisoned patients. **Table S2.** Propensity score used in the multivariate analysis to establish the contribution of L-carnitine to valproic acid-poisoned patient management. **Table S3.** Multivariate analysis evaluating the effect of L-carnitine administration on the outcome of valproic acid-poisoned patients.

## Data Availability

The data set used during the current study are available from the corresponding author on reasonable request.

## Abbreviations

VPA: Valproic acid
ICU: Intensive care unit
SAPS II: Simplified Acute Physiology Score II
SOFA: Sequential Organ Failure Assessment
*t*_1/2_: Elimination half-life
