# Supplementary material for: L-carnitine does not improve valproic acid poisoning management: a cohort study with toxicokinetics and concentration/effect relationships
Source: Ann Intensive Care. 2022 Jan 29;12:7. doi: 10.1186/s13613-022-00984-z (PMC8800998; doi:10.1186/s13613-022-00984-z)
Supplement: Supplementary file 1 — Additional file 1: Table S1. Descriptive analysis of different subgroups of valproic acid-poisoned patients. Table S2. Propensity score used in the multivariate analysis to establish the contribution of L-carnitine to valproic acid-poisoned patient management. Table S3. Multivariate analysis evaluating the effect of L-carnitine administration on the outcome of valproic acid-poisoned patients. [file 13613_2022_984_MOESM1_ESM.docx]

**Table S1** Descriptive analysis of different subgroups of valproic acid-poisoned patients

|  | **On admission** | | | | **During ICU stay** | | | | **Outcome** |
| --- | --- | --- | --- | --- | --- | --- | --- | --- | --- |
|  | Arterial pH | Blood lactate level (mmol/L) | VPA concentration (mg/L) | Ammonemia (mmol/L) | L-carnitine infusion | Activated charcoal | ICU management | Complications |  |
| **Brain edema**  *Patient 19*  *Patient 62* | 7.41  7.46 | 2.6  3.8 | 494  197 | 75  166 | Yes  Yes | No  Yes | MV, NE  MV, NE | Co, Sh, Tcp, An, AP  Co, Sh, Tcp, An, AP | Home  Home |
| **Lactic acidosis**  *Patient 14*  *Patient 25*  *Patient 31*  *Patient 34*  *Patient 39*  *Patient 40*  *Patient 54*  *Patient 56*  *Patient 60* | 7.30  6.99  7.30  7.29  7.25  7.33  7.33  7.32  7.18 | 6.5  19.0  5.6  5.9  6.1  4.6  5.7  7.2  9.2 | 304  180  887  654  42  167  273  93  269 | -  -  -  -  -  -  -  -  - | No  Yes  Yes  No  No  No  No  No  Yes | No  Yes  Yes  No  No  Yes  Yes  No  No | MV, NE  MV, NE, HD  MV, NE  NIMV  MV  MV, NE  NIMV  NIMV  MV | Co, Sh, AP  Co, Sh, AKI, LC, AP  Co, Sh, AP, DIVC  Tachy, AKI  Co  Co, Sh, Tcp, AP  Co  Tachy  Co, AKI | Psychiatric ward  Death  Death  Psychiatric ward  Home  Medical ward  Home  Home  Psychiatric ward |
| **Hyperammonemia**  *Patient 03*  *Patient 10*  *Patient 19*  *Patient 16*  *Patient 33*  *Patient 62*  *Patient 65*  *Patient 73* | 7.40  7.41  7.41  7.39  7.42  7.46  7.36  7.43 | 2.0  2.7  2.6  4.1  2.3  3.8  4.1  4.6 | 234  140  494  278  375  197  820  101 | 124  140  75  278  375  197  96  91 | No  No  Yes  Yes  No  Yes  No  Yes | No  No  No  No  Yes  Yes  Yes  No | None  None  MV, NE  None  None  None  None  MV | Agi  Agi  Co, Sh, Tcp, An, AP  Tachy  None  Co, Sh, Tcp, An, AP  An  Co | Psychiatric ward Home  Home  Home  Psychiatric ward Home  Home  Medical ward |
| **Very elevated initial VPA concentration**  *Patient 06*  *Patient 09*  *Patient 12*  *Patient 17*  *Patient 19*  *Patient 31*  *Patient 34*  *Patient 43*  *Patient 47*  *Patient 52*  *Patient 59*  *Patient 65*  *Patient 70* | 7.42  7.44  7.37  7.36  7.41  7.30  7.29  7.43  7.43  7.37  7.37  7.36  7,42 | 2.2  3.8  2.7  3.0  2.6  5.6  5.9  3,4  6.2  4.3  3.3  4.1  4,2 | 633  692  849  1,070  494  887  654  535  1,264  569  1,301  820  719 | -  -  -  -  75  -  -  -  -  -  -  96  - | No  Yes  Yes  Yes  Yes  Yes  No  No  No  Yes  Yes  No  No | No  Yes  No  Yes  No  Yes  No  No  No  Yes  No  Yes  Yes | MV, NE  MV  MV, NE  MV  MV, NE  MV, NE  NIMV  MV  MV  MV  MV  None  IV | Agi., Sh, Tcp, AP  Agi, Aki, AP  Sh, AP  AKI, AP  Co, Sh, Tcp, An, AP  Co, Sh, AP, DIC  Tachy, AKI  None  Co, AP  Sh, Tcp, An, AKI  Co, Tcp, An  An  Tachy, AKI, AP | Psychiatric ward Psychiatric ward  Psychiatric ward Psychiatric ward  Home  Death  Psychiatric ward Medical ward  Medical ward  Medical ward  Psychiatric ward Home  Psychiatric ward |

Agi, agitation; AKI, acute kidney injury; An, anemia; AP, aspiration pneumonia; Co, coma; DIC, Disseminated intravascular coagulation; HD, hemodialysis; LC, liver cytolysis; MV, invasive mechanical ventilation; NIMV, noninvasive mechanical ventilation; NE, norepinephrine; Sh, shock; Tachy, tachycardia; Tcp, thrombocytopenia. Lactic acidosis was defined as arterial pH <7.35 and blood lactate level >2.0 mmol/L. Hyperammonemia was defined as blood ammonia concentration > 55 mmol/L. Very elevated VPA concentration on admission was defined as >450 mg/mL.

**Table S2.** Propensity score used in the multivariate analysis to establish the contribution of L-carnitine to valproic acid-poisoned patient management

|  | **Univariate analysis** | | | **Multivariate analysis (logistic regression)** | |
| --- | --- | --- | --- | --- | --- |
|  | **Patients managed without L-carnitine (n=50)** | **Patients managed with L-carnitine (n=19)** | ***P*-value** | **Odds ratio [95% confidence interval]** | ***P*-value** |
| Age | 40 [34-47] | 40 [29-48] | 0.70 | - | NS |
| Gender (F/M) | 29 (58%) / 21 (42%) | 11 (58%) / 8 (42%) | 1.00 | - | NS |
| Plasma VPA concentration on ICU admission (mg/L) | 210 [143-358] | 274 [174-607] | 0.11 | - | NS |
| Blood lactate concentration on ICU admission (mmol/L) | 2.7 [1.7-4.1] | 3.3 [2.0-4.7] | 0.001 | 1.31 [0.98-1.75] | 0.07 |
| SOFA score on ICU admission | 4 [2-6] | 4 [1-5] | 0.59 | - | NS |

ICU, intensive care unit; SOFA, Sequential Organ Failure Assessment; VPA, valproic acid

This table shows how the propensity score was built. The detailed univariate and multivariate analyzes are presented in Table 3S. Among parameters included in the model, only blood lactate level on admission was associated with L-carnitine administration in univariate (*P* = 0.001), by contrast to age, gender, SOFA score, and VPA concentration on admission. The propensity score used in the following analyses as covariate associated with L-carnitine was calculated according to the following equation: Score = -2.157+ (0.271 * blood lactate level on admission (mmol/L).

**Table S3.** Multivariate analysis evaluating the effect of L-carnitine administration on the outcome of valproic acid-poisoned patients

| **Variable** | **Estimated parameter** | **OR [95% IC]** | ***P*-value** |
| --- | --- | --- | --- |
| Constant | -2.482 |  |  |
| Peak blood lactate level (mmol/L) | 0.617 | 1.9 [1.2-2.8] | 0.004 |

The following parameters were included in the model: the Glasgow coma score on admission, the blood lactate level on admission, the plasma valproic acid on admission, the peak blood lactate, the delay of blood lactate level normalization, the presence of consciousness impairment, and the onset of brain edema. Only the peak lactate level was significantly correlated with L-carnitine administration.
